# Cries, Stridor, and Clues: Unmasking a Rare Airway Obstruction in an Infant

**DOI:** 10.7759/cureus.101842

**Published:** 2026-01-19

**Authors:** Karthikeyan V P, Dharshana R, Hariprasad R, Senthil Kumar K, Senguttuvan D, Hari Meyyappan

**Affiliations:** 1 Pediatrics, Sinduja Hospital, Trichy, IND; 2 Otolaryngology-Head and Neck Surgery, KMC Speciality Hospitals (India) Ltd, Trichy, IND; 3 Pediatrics, KMC Speciality Hospitals (India) Ltd, Trichy, IND

**Keywords:** airway obstruction, laryngomalacia, respiratory distress, stridor, vallecular cyst

## Abstract

Laryngomalacia is the predominant cause of airway obstruction resulting in stridor among newborns, constituting a significant percentage of all congenital stridor instances. Vallecular cysts, though rare, represent an important differential diagnosis, responsible for a notable percentage of all congenital laryngeal cysts. The frequency of vallecular cysts is considered low among newborns. We report a vallecular cyst case in a baby who had respiratory distress and inspiratory stridor. Diagnosis was made by direct laryngoscopy, and the cyst was completely excised, resulting in the resolution of symptoms. On follow-up after a few months, there was no evidence of recurrence. This case is documented due to its rarity and to enhance awareness of vallecular cysts as a possible etiology of stridor and respiratory distress in babies. It also emphasizes the importance of thorough clinical assessment and interdisciplinary evaluation. Furthermore, the case highlights the effectiveness of complete excision over marsupialization in preventing recurrence.

## Introduction

Vallecular cysts are one of the rare causes of respiratory distress and inspiratory stridor in infancy [1]. Feeding difficulty, inspiratory stridor, noisy breathing, respiratory distress, and failure to thrive are common presentations in infants. It is found to be associated with laryngomalacia [2]. We describe a case of vallecular cyst in an infant who presented with an inspiratory stridor that was diagnosed by direct laryngoscopy and treated with an excision of the cyst leading to the complete resolution of the stridor. Though it is a benign cyst, if not identified early, it would lead to catastrophic complications and even be life-threatening [3]. This case report highlights the significance of a comprehensive clinical and interdisciplinary evaluation of neonatal stridor for an early identification and intervention, in order to prevent catastrophic consequences and potentially fatal results.

## Case presentation

A three-month-old baby girl, first born out of a non-consanguineous marriage, was referred to our hospital with noisy breathing for two months, which got exaggerated and associated with respiratory distress for the past 10 days before admission. She was born at term by lower segment caesarean section (LSCS) with an uneventful neonatal period and managed elsewhere as lower respiratory infection with laryngomalacia. With increasing respiratory distress and poor feeding with elevated ammonia levels (>7000), the baby was referred to our hospital.

**Figure 1:**
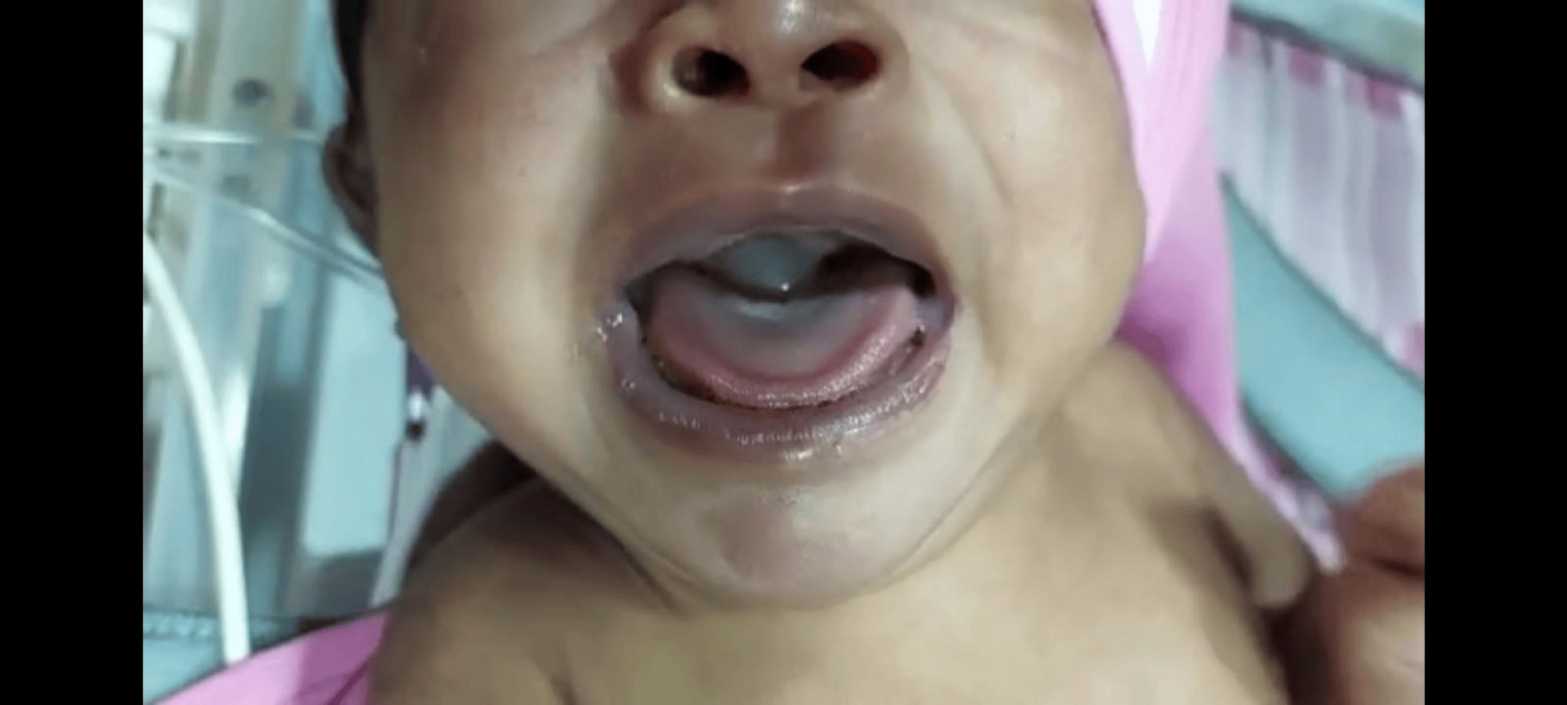
Cystic mass seen at the base of the tongue while crying

Upon examination, her vitals were stable, and she was in respiratory distress with marked subcostal, suprasternal retractions with a Downes score of 3/10. Oxygen supplementation was started. Air entry was equal on both sides with inspiratory stridor. Other systems examinations were normal. Laboratory investigations done to rule out infection were within normal limits (Table 1). Serum ammonia repeated in our hospital was normal. Arterial blood gas analysis was normal (Table 2). During crying, we noticed a cystic mass at the base of the tongue (Figure 1). Direct laryngoscopy revealed a well-circumscribed cystic mass at the base of the tongue obstructing the laryngeal inlet. The base of the lesion could not be made out.

**Table 1:** Blood investigations WBC: white blood cell; RBC: red blood cell; PCV: packed cell volume; MCV: mean corpuscular volume; MCHC: mean corpuscular hemoglobin concentration; ESR: erythrocyte sedimentation rate; CRP: C-reactive protein

Investigation	Result	Reference range
Hemoglobin	9.7 g/dL	9.4-13.5 g/dL
Total WBC	11000 cells/mm^3^	6000-18000 cells/mm^3^
Lymphocytes	43.1%	55-65%
Basophil	1%	0-2%
Monocytes	10.6%	1-2%
Eosinophil	1.8%	1-6%
RBC count	3.15×10^6^/mm^3^	3.60×10^6^/mm^3^
PCV	28.7%	28-42%
MCV	30.7 fL	68-105 fL
MCHC	33.7 g/dL	28.0-38.0 g/dL
Platelet count	483×10^3^/mm^3^	150-400×10^3^/mm^3^
ESR	2 mm/1 hour	3-13 mm/1 hour
CRP	3.75 mg/dL	0-5 mg/dL
Sodium	138.8 mmol/L	136-145 mmol/L
Potassium	4.28 mmol/L	3.5-5.1 mmol/L
Chloride	105 mmol/L	98-107 mmol/L
Bicarbonate	22.7 mmol/L	22-26 mmol/L
Ammonia	57 µmol/L	Up to 50 µmol/L
Calcium	9.54 mg/dL	8.0-11.3 mg/dL

**Table 2:** Arterial blood gas report pCO2: partial pressure of carbon dioxide; PO2: partial pressure of oxygen; HCO3: bicarbonate; CO2: carbon dioxide; O2: oxygen

	Result	Reference range
Blood gas pH	7.37	7.34-7.44
Blood gas pCO2	30 mmHg	35-45 mmHg
Blood gas PO2	165 mmHg	75-100 mmHg
Blood gas HCO3	19.4 mmol/L	22-26 mmol/L
Blood gas base excess	-4.4	0-2.3
Blood gas total CO2	18.2 mmol/L	23-27 mmol/L
Blood gas O2 saturation	99.5%	95-100%

**Figure 2:**
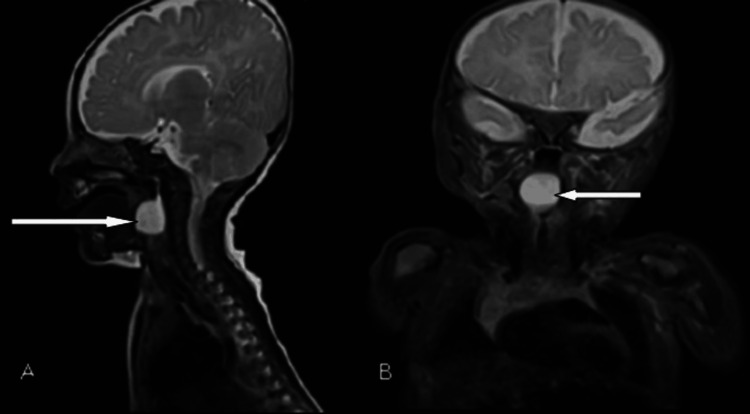
MRI sagittal (A) and coronal (B) section showing a well-defined rounded cystic lesion measuring 16×14 mm with broad base attachment abutting the posterior pharyngeal wall causing the almost complete airway obstruction (white arrow) MRI: magnetic resonance imaging

Magnetic resonance imaging (MRI) of the neck was performed after stabilizing the baby and showed a well-defined rounded cystic lesion measuring 16×14 mm with a broad base attachment abutting the posterior pharyngeal wall causing the almost complete obstruction of the airway (Figure 2). Vallecular cyst or saccular cyst was kept as a differential diagnosis based on MRI findings.

**Figure 3:**
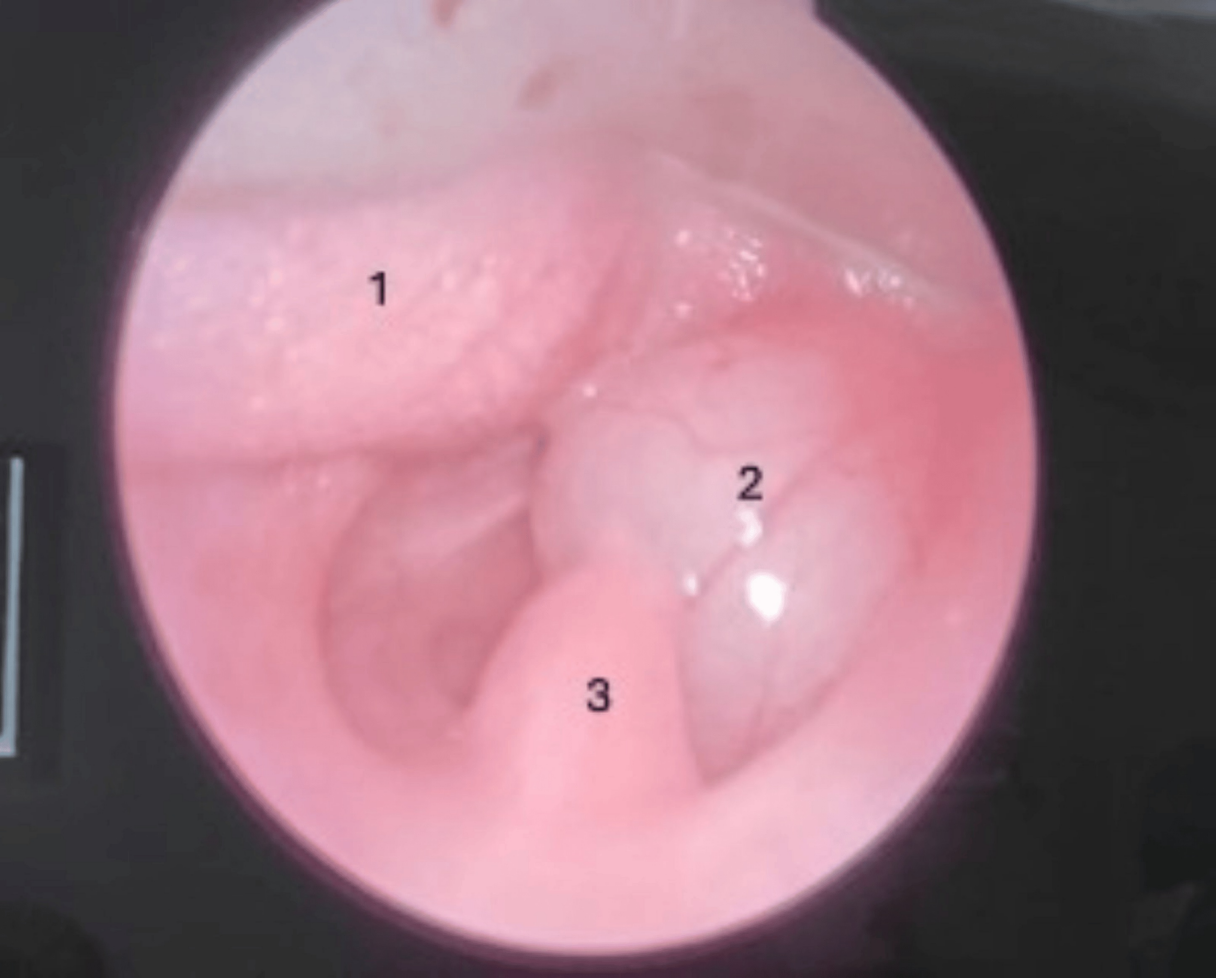
Laryngoscopic view of the vallecular cyst attached to the right lateral pharyngeal wall, right vallecula, aryepiglottic fold, and right lateral wall of the epiglottis. (1) Base of the tongue, (2) vallecular cyst, and (3) uvula

**Figure 4:**
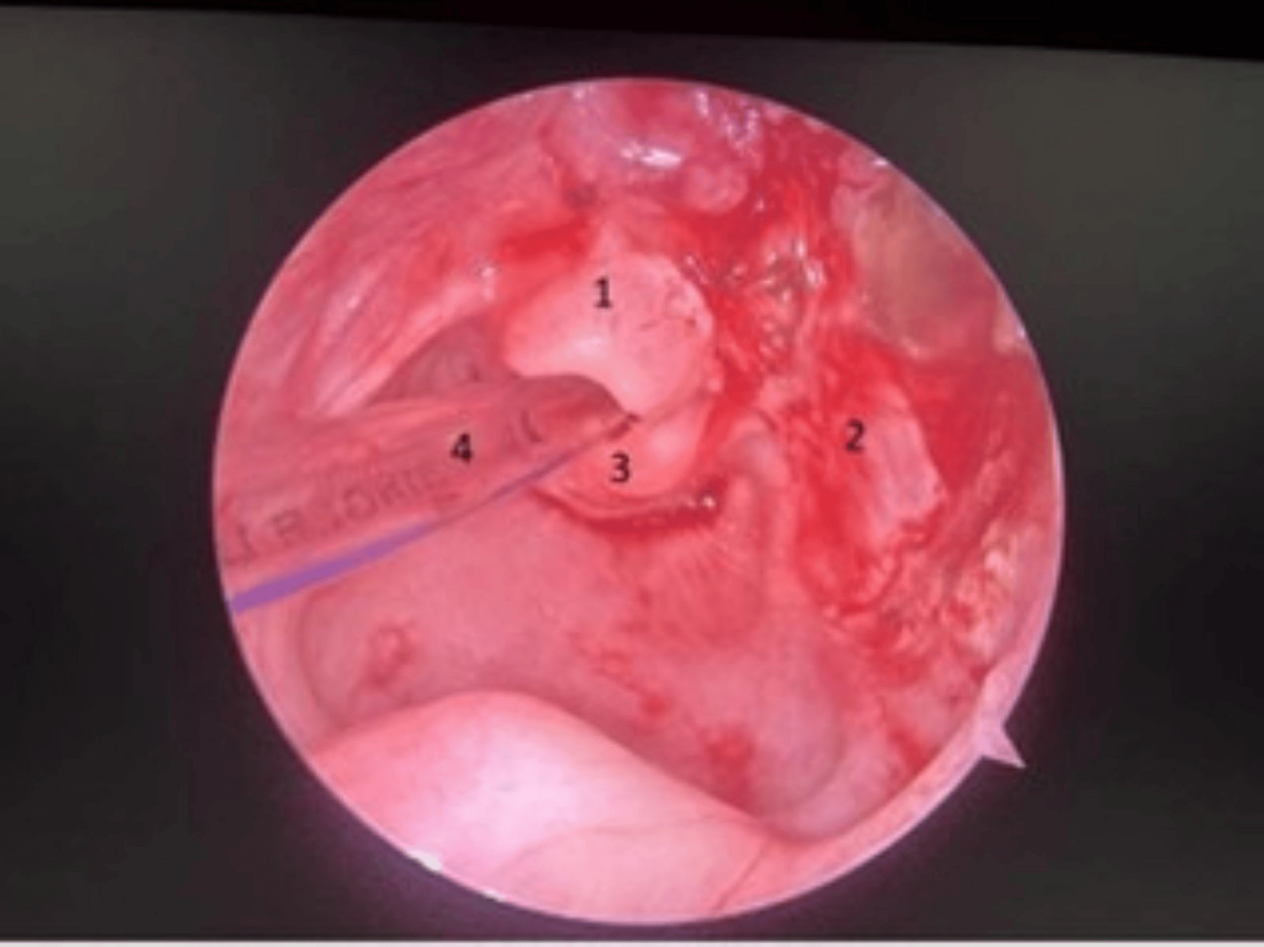
Postoperative laryngoscopic view of the site of the vallecular cyst showing complete resolution of the cyst and patent airway. (1) Epiglottis, (2) base of the cyst (right lateral pharyngeal wall), (3) arytenoid, and (4) endotracheal tube

The cyst was examined under endoscopy, which was attached to the right lateral pharyngeal wall, right side vallecula, and right lateral wall of the epiglottis. The supraglottic space was found free. The cyst contained a clear mucoid fluid that was aspirated, and the airway was secured with endotracheal intubation. Then, the cyst was excised in toto under general anesthesia and sent for biopsy. A final diagnosis of a vallecular cyst was made based on MRI and per operative findings (Figure 3 and Figure 4). The postoperative period was uneventful. The baby was on nasogastric feeds initially and was put on direct breastfeeding on postoperative day 2. Respiratory distress was remarkably improved, and the patient was discharged.

## Discussion

Stridor in a neonate potentially implies that the airway is compromised, necessitating an appropriate intervention. Because of a narrower airway, small changes in airway diameter can cause a considerable increase in airway resistance [4]. Stridor caused by obstruction in the extrathoracic area is more noticeable during inhalation, resulting in an inspiratory stridor. In contrast, lesions in the intrathoracic region lead to an expiratory stridor. It may result from various conditions that can be either congenital or acquired. Even an upper respiratory infection can manifest as acute stridor. The intricate structure of the larynx, with its many folds and outpouchings, makes this area susceptible to congenital malformations. The age at which stridor begins is the main factor that can assist in determining its cause. Extrathoracic causes of stridor are laryngomalacia, vocal cord paralysis, congenital structural abnormalities, infections like epiglottitis, thyroglossal cyst, subglottic stenosis, cysts, and hemangioma [5]. Intrathoracic causes of expiratory stridor may include tracheobronchomalacia, tracheal stenosis, vascular ring, and foreign body [6]. A biphasic stridor is due to severe laryngomalacia, tracheomalacia, or vocal cord paresis or less often caused by subglottic hemangioma or vascular ring [7]. While most congenital airway anomalies manifest within the first few weeks of life, some anomalies, like bronchogenic cysts and laryngeal clefts, may appear later in infancy or childhood. In infants with dysmorphism and stridor, a complete examination of the nose and oropharynx is essential to assess for structural abnormalities like choanal atresia, micrognathia, macroglossia, and craniofacial abnormalities.

Laryngomalacia is the most common cause of chronic extrathoracic airway obstruction, leading to stridor in newborns, affecting 45-75% of all infants with congenital stridor [8]. It is typically present in the neonatal period and progresses during infancy but is expected to resolve within 12-18 months. It appears as inspiratory stridor, which tends to worsen when in a supine position and during feeding and sleeping. Stridor becomes exaggerated during upper respiratory tract infections. It is distinct from tracheomalacia which usually presents with expiratory or biphasic stridor.

Congenital vallecular cysts are uncommon, representing 10-20% of all congenital laryngeal cysts [9]. The overall incidence rate of vallecular cysts is around 3.49-5.3 cases per 100,000 newborns [10]. It consists of respiratory epithelium and mucous glands on histological examination. They develop in the hypopharynx, between the epiglottis and the base of the tongue. It has been known as a mucous retention cyst, epiglottic cyst, base-of-the-tongue cyst, and ductal cyst as per the classification of laryngeal cyst by Desanto et al. [11]. They are secondary cysts formed from either the ductal obstruction of mucous glands or cystic tongue lesions formed from misplaced embryonic remnants of the foregut [12].

Aryepiglottic cysts are the most common laryngeal cysts in children, followed by vallecular cysts, which are unilocular cysts containing clear mucous sterile fluid arising from the lingual surface of the epiglottis [13]. Feeding difficulties, failure to thrive, inspiratory stridor, noisy respiration, respiratory distress, and a hoarse cry are the common presentations in infants with vallecular cysts. Laryngomalacia is an associated finding. Diagnosis of vallecular cysts warrants a high level of clinical suspicion. Nasopharyngoscopy, laryngoscopy, and bronchoscopy provide a visualization of the airways, facilitating a definitive diagnosis of the cause of stridor in children. More than one airway abnormality may be present in the same child, and a thorough evaluation of the upper and lower airways may be warranted. MRI is helpful in the preoperative period to delineate the cyst's structure, vascularity, and extension and can identify other associated malformations [14]. Other cystic lesions that can cause stridor, such as lingual thyroid, thyroglossal cyst, and lymphatic malformation, should also be considered in the differential diagnosis of a vallecular cyst. In our case, we did a direct laryngoscopy and MRI for diagnosis.

Definitive treatment includes marsupialisation. This can be done either transorally using microscissors or a laser or via a transhyoid resection [15]. Aspiration or rupture may provide temporary improvement in symptoms but increases the risk of cyst recurrence, but it can be considered in emergencies, such as severe airway obstruction, before attempting the complete removal of the cyst as we did in our case. The cyst content was aspirated, and the airway was secured with an endotracheal tube, followed by the excision of the cyst which showed a significant improvement in symptoms with no postoperative complications.

## Conclusions

Vallecular cysts, though rare, are a critical differential diagnosis in infants presenting with stridor, respiratory distress, and feeding difficulties. Early diagnosis and prompt intervention are essential to prevent life-threatening airway obstruction. This case highlights the importance of maintaining a high index of suspicion for congenital anomalies such as vallecular cysts in neonates with persistent or worsening stridor, especially when initial treatments for more common conditions like laryngomalacia fail to provide relief. Comprehensive clinical evaluation, supported by direct laryngoscopy and imaging studies like MRI, is crucial for accurate diagnosis and surgical planning.

Definitive surgical excision remains the treatment of choice, providing excellent outcomes and preventing recurrence. In this case, endoscopic excision following initial aspiration ensured airway security and resulted in the complete resolution of symptoms without complications. The case underscores the need for interdisciplinary collaboration involving pediatricians, radiologists, and otolaryngologists for timely diagnosis and management. Educating clinicians about such rare but significant causes of neonatal stridor can aid in early identification and reduce the risk of severe respiratory compromise.
